# Supplementary material for: Reversal of transmission and reflection based on acoustic metagratings with integer parity design
Source: Nat Commun. 2019 May 24;10:2326. doi: 10.1038/s41467-019-10377-9 (PMC6534607; doi:10.1038/s41467-019-10377-9)
Supplement: Supplementary file 1 — Supplementary Information [file 41467_2019_10377_MOESM1_ESM.pdf]

Supplementary Information for

"Reversal of transmission and reflection based on acoustic metagratings with integer parity design"

Yangyang Fu[1, 3] , Chen Shen[2], Yanyan Cao[1], Lei Gao[1], Huanyang Chen[5], C. T. Chan[4], Steven A. Cummer[2] & Yadong Xu[1]

[1] *School of Physical Science and Technology & Jiangsu Key Laboratory of Thin Films, Soochow University, Suzhou 215006, China.*

[2] *Department of Electrical and Computer Engineering, Duke University, Durham, North Carolina 27708, America.*

[3] *College of Science, Nanjing University of Aeronautics and Astronautics, Nanjing 211106, China.*

[4] *Department of Physics, Hong Kong University of Science and Technology, Clear Water Bay, Hong Kong, China.*

[5] *Institute of Electromagnetics and Acoustics and Key Laboratory of Electromagnetic Wave Science and Detection Technology, Xiamen University, Xiamen 361005, China.*

Supplementary Figures 1 to 7
Supplementary Notes 1 to 6
Supplementary References

Supplementary Figures

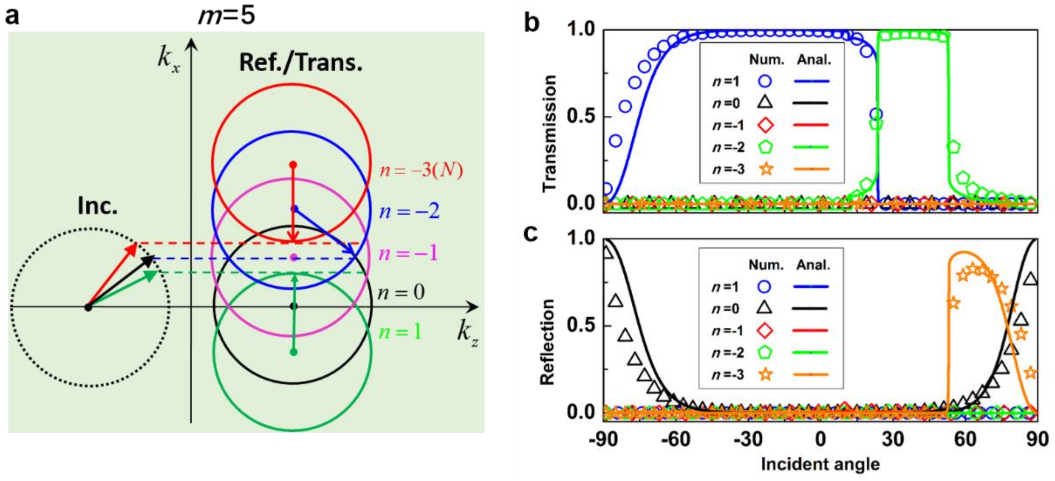

Supplementary Figure 1. *m*=5 case. **a,** The equi-frequency contour of PGM with $\xi = 0.6k_0$. **b, c** are the corresponding relationships between transmission and reflection of the diffraction orders and incident angle, respectively, where the number of unit cells is $m = 5$. The gradient index materials in unit cells are impedance matched ($n_i = \rho_i$) and the filling rate in each cell is $f = w/a = 0.9$.

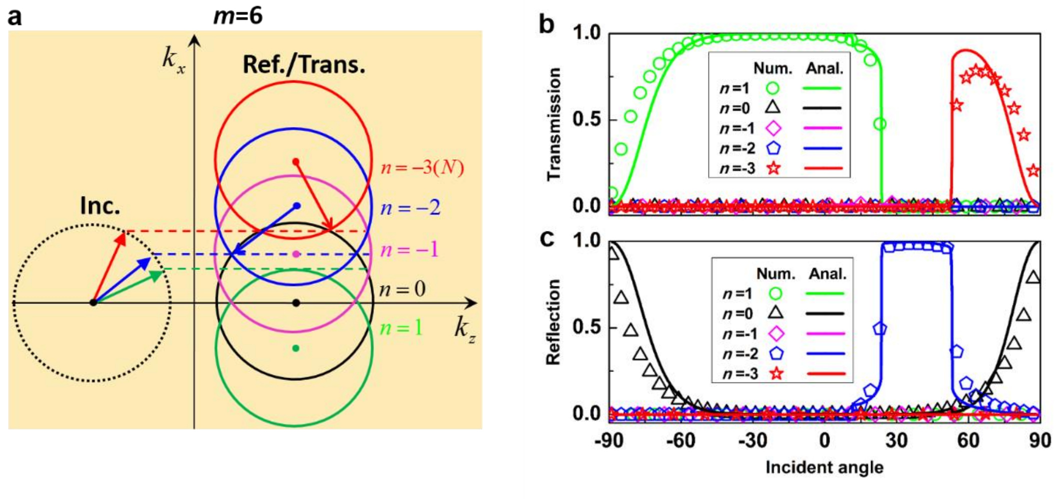

Supplementary Figure 2. *m*=6 case. **a**, The equi-frequency contours of GIM with $\xi = 0.6k_0$. **b, c** are the corresponding relationships between transmission and reflection of the diffraction orders and incident angle, respectively, where the number of unit cells is $m = 6$. The gradient index materials in unit cells are impedance matched ($n_i = \rho_i$), and the filling rate in each cell is $f = w/a = 0.9$.

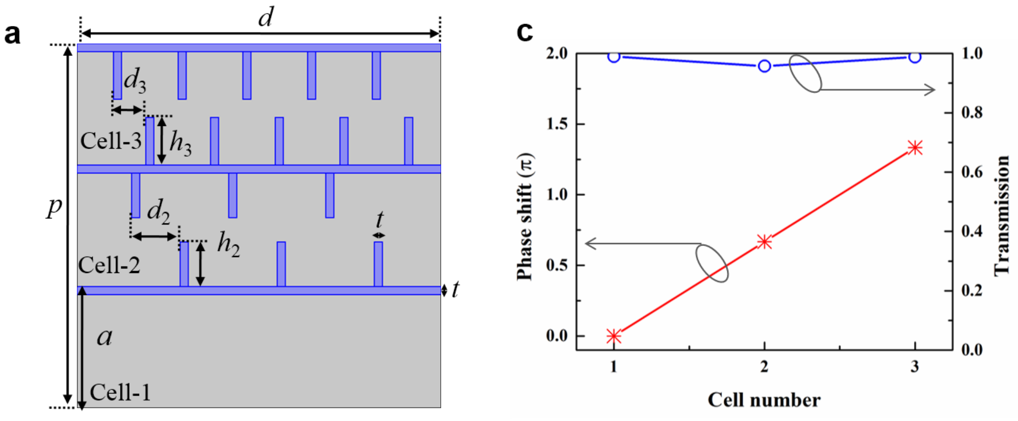

b

Number	Distance of Blocks	Height of Blocks	Constants
Cell-1	0	0	t=1.905mm
Cell-2	d_2=6t	h_2=0.39(a-t)	a=28.583mm
Cell-3	d_3=4t	h_3=0.42 (a-t)	p=d=85.750mm

Supplementary Figure 3. The design detail for PGM ($m=3$) with $\xi = k_0$. **a**, Geometric topography of a supercell. **b**, Designed parameters for the three different unit cells. **c**, Phase shift (left) and transmission (right) of the three different unit cells.

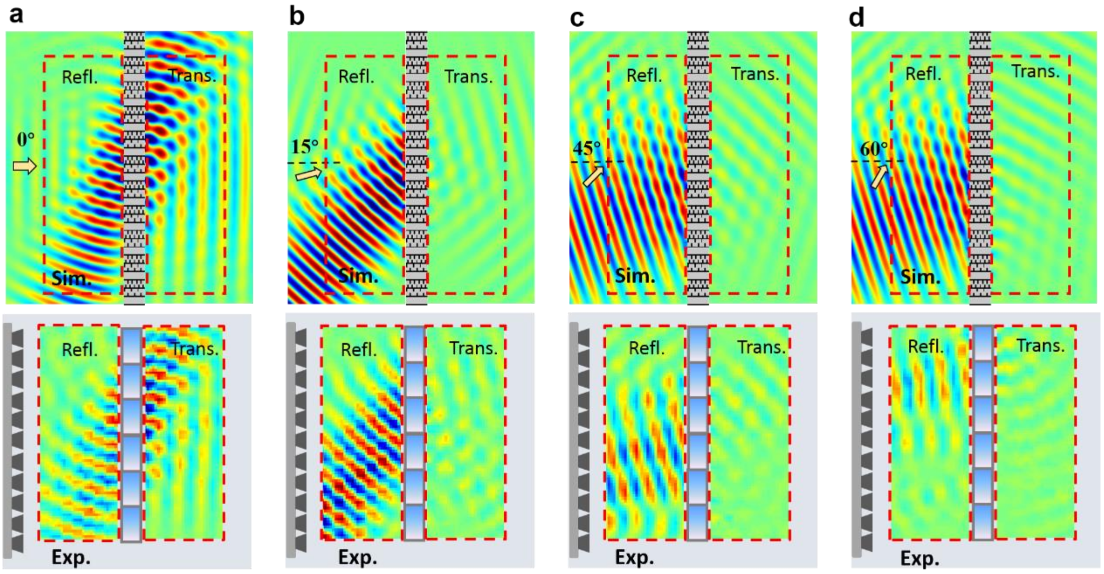

Supplementary Figure 4. The numerically simulated (upper) and experimentally measured (lower) acoustic pressure field for the designed PGM ($m=3$) with $\xi = k_0$. **a**, $\theta_{\text{in}} = 0^\circ$. **b**, $\theta_{\text{in}} = 15^\circ$. **c**, $\theta_{\text{in}} = 45^\circ$. **d**, $\theta_{\text{in}} = 60^\circ$.

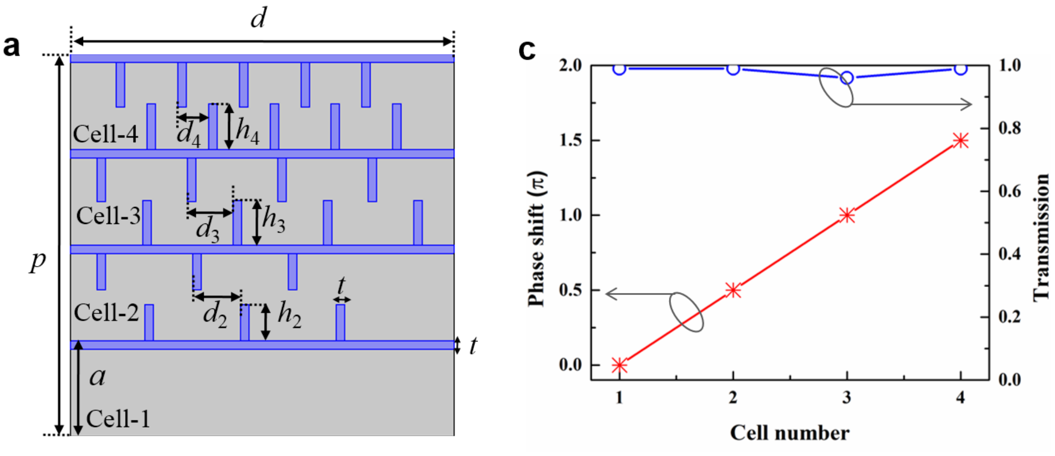

b

Number	Distance of Blocks	Height of Blocks	Constants
Cell-1	0	0	t=1.905mm
Cell-2	d_2=5.6t	h_2=0.415(a-t)	a=21.4375mm
Cell-3	d_3=5.3t	h_3=0.505 (a-t)	p=d=85.750mm
Cell-4	d_4=3.6t	h_4=0.520 (a-t)	

Supplementary Figure 5. The design detail for PGM ($m = 4$) with $\xi = k_0$. **a**, Geometric topography of a supercell. **b**, Designed parameters for the four different unit cells. **c**, Phase shift (left) and transmission (right) of the four different unit cells.

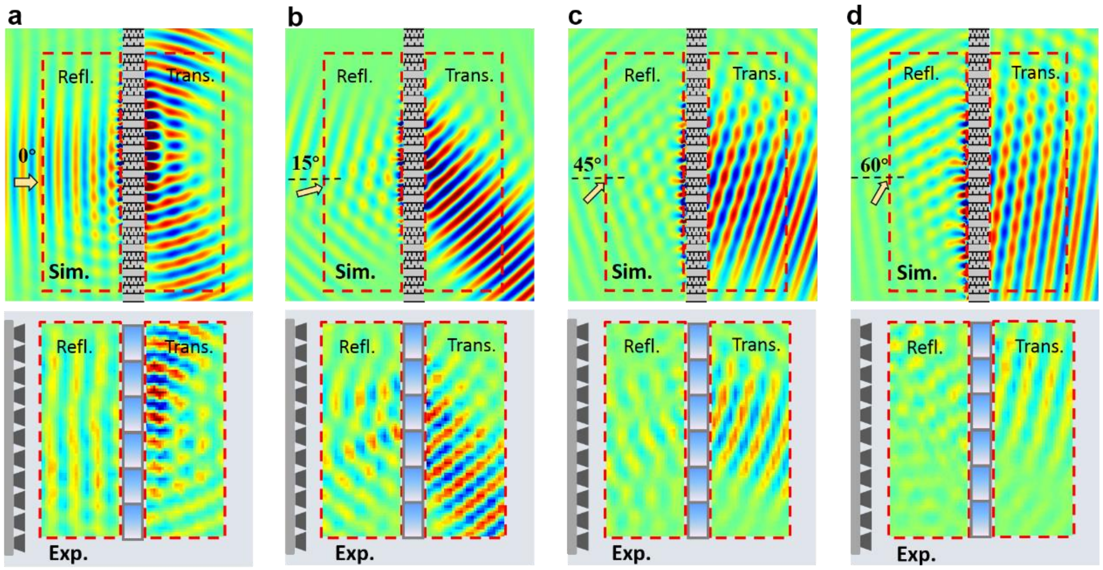

Supplementary Figure 6. The numerically simulated (upper) and experimentally measured (lower) acoustic pressure field for the designed PGM ($m = 4$) with $\xi = k_0$. **a**, $\theta_{\rm in} = 0^\circ$. **b**, $\theta_{\rm in} = 15^\circ$. **c**, $\theta_{\rm in} = 45^\circ$. **d**, $\theta_{\rm in} = 60^\circ$.

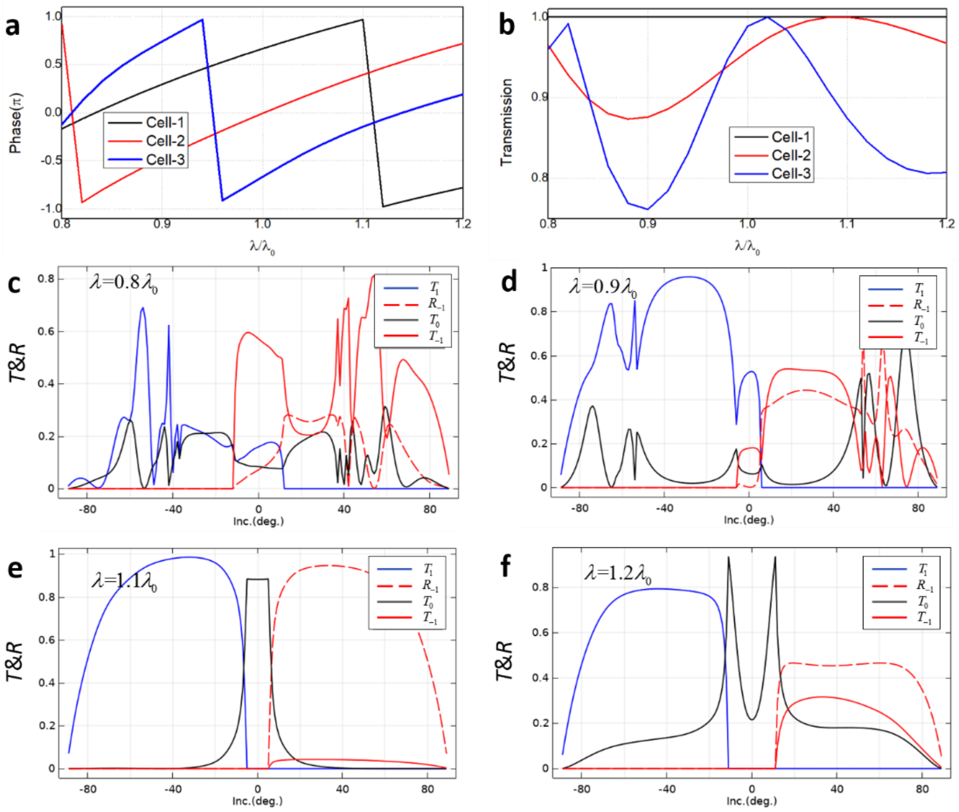

Supplementary Figure 7. Performance of the designed PGM ($m = 3$) in a broadband of frequencies. **a**, Phase response of three unit cells. **b**, Transmission response of three unit cells. Transmission and reflection at different wavelengths (**c**-**f**). **c**, λ=0.8λ_0 . **d**, λ=0.9λ_0 . **e**, λ=1.1λ_0 . **f**, λ=1.2λ_0 .

Supplementary Notes

Supplementary Note 1. Coupled mode theory for the acoustic phase gradient metagrating

As shown in Fig. 1(a), the proposed acoustic metagrating is composed of *m* unit cells with widths of *a*. The periodic length is $p = ma$ and the thickness of the grating is *h*. Each unit cell is perforated by a slit with a width of *w*. Each period with *m* individual unit cells should cover 2π abrupt phase change, and the phase difference of two adjacent unit cells is $\Delta\phi = 2\pi / m$. To achieve that, these unit cells are filled with impedance matched material, i.e., $\rho_j = n_j = 1 + (j-1)\lambda / mh$, where $j = 1, 2 \ldots m$. When acoustic wave with an incident angle of θ_{in} bumps on the phase gradient metagrating (PGM), the entire system is divided into three regions: the incident region (region 1), the PGM region (region 2) and the transmitted region (region 3). In the incident side, the total pressure field is the superposition of incident wave and the reflected waves of all the diffraction orders,

$$P_1 = \sum_n \left[\delta_{n,0} \exp(ik_z^n z) + r_n \exp(-ik_z^n z) \right] \exp(i\alpha_n x), \tag{S1}$$

where $\delta_{n,0}$ is the Kronecker delta, $k_z^n = \sqrt{k_0^2 - \alpha_n^2}$, $\alpha_n = k_0 \sin\theta_{\text{in}} + nG$ and $G = 2\pi / p$, with $k_0 = 2\pi / \lambda$ is the wavevector in free space; r_n is the reflection coefficient of the *n*-th diffraction order. In outgoing side, the transmitted wave includes all the transmission components of diffraction orders, *i.e.*,

$$P_3 = \sum_n t_n \exp\left[ik_z^n (z-h) \right] \exp(i\alpha_n x), \tag{S2}$$

where t_n is the transmission coefficient of the *n*-th diffraction order. In the grating region, as the width of unit cell is considerably smaller than the operating wavelength, only the fundamental mode is considered. As a result, acoustic wave in unit cell is the standing wave, i.e.,

$$P_{2,j} = a_j \exp(ik_j z) + b_j \exp(-ik_j (z-h)), \quad (j = 1, 2 \ldots m), \tag{S3}$$

where a_j and b_j are the amplitude coefficients of the forward and backward waves and $k_j = n_j k_0$ is the wave vector in the *j*-th unit cell. Based on $-jw\rho v_z = \partial P / \partial z$, the corresponding *z*-components of the velocity fields are written as,

$$v_{1z} = \frac{1}{\omega\rho_0} \sum_n k_z^n \left[\delta_{n,0} \exp(ik_z^n z) - r_n \exp(-ik_z^n z) \right] \exp(i\alpha_n x), \tag{S4}$$

$$v_{2z,j} = \frac{k_j}{\omega\rho_j} [a_j \exp(ik_j z) - b_j \exp(-ik_j (z-h))], \quad (x \le x_j + |w/2|) \tag{S5a}$$

$$v_{2z,j} = 0, \quad (|w/2| \le x - x_j \le |a/2|) \tag{S5b}$$

$$v_{3z} = \frac{1}{\omega\rho_0} \sum_n k_z^n t_n \exp\left[ik_z^n (z-h) \right] \exp(i\alpha_n x), \tag{S6}$$

where x_j=$(j-1)a$ and ρ_0 is mass density in free space. By applying the continuous boundary conditions of pressure field and velocity field at *z*=0 and *z*=*h*, we have

$$\sum_n (\delta_{n,0}+r_n)\exp(i\alpha_n x)=a_j+b_j u_j, \quad (x\le x_j+|w/2|) \tag{S7}$$

$$\sum_n k_z^n\left[\delta_{n,0}-r_n\right]\exp(i\alpha_n x)/\rho_0=\frac{k_j}{\rho_j}(a_j-b_j u_j), \quad (x\le x_j+|w/2|) \tag{S8a}$$

$$\sum_n k_z^n\left[\delta_{n,0}-r_n\right]\exp(i\alpha_n x)/\rho_0=0, \quad (|w/2|\le x-x_j\le|a/2|) \tag{S8b}$$

$$\sum_n t_n\exp(i\alpha_n x)=a_j u_j+b_j, \qquad (x\le x_j+|w/2|) \tag{S9}$$

$$\sum_n t_n k_z^n\exp(i\alpha_n x)/\rho_0=\frac{k_j}{\rho_j}(a_j u_j-b_j), \quad (x\le x_j+|w/2|) \tag{S10a}$$

$$\sum_n t_n k_z^n\exp(i\alpha_n x)/\rho_0=0, \quad (|w/2|\le x-x_j\le|a/2|) \tag{S10b}$$

where $u_j=\exp(ik_j h)$. By integrating the Eq. (S7) and Eq. (S9) with *dx* at the region $x\le x_j+|w/2|$, we can get,

$$\sum_n g_n(\delta_{n,0}+r_n)\exp(i\alpha_n x_j)=a_j+b_j u_j, \tag{S11}$$

$$\sum_n g_n t_n\exp(i\alpha_n x_j)=a_j u_j+b_j, \tag{S12}$$

where $g_n=\mathrm{Sin}c(\alpha_n w/2)$. Furthermore, by multiplying both sides of Eq. (S8) and Eq. (S10) with $\exp(-i\alpha_n x)$, and then integrating both sides with *dx* at the region $0\le x\le p$, we can obtain,

$$\frac{k_z^n}{\rho_0}(\delta_{n,0}-r_n)=\sum_{j=1}^{m}\frac{k_j}{\rho_j}g_n f(a_j-b_j u_j)\exp(-i\alpha_n x_j), \tag{S13}$$

$$\frac{t_n k_z^n}{\rho_0}=\sum_{i=1}^{m}\frac{k_j}{\rho_j}fg_n(a_j u_j-b_j)\exp(-i\alpha_n x_j), \tag{S14}$$

where $f=w/p$ with $p=ma$. By solving Eq. (S11) through Eq. (S14), we can analytically obtain all unknown coefficients of r_n and t_n.

Supplementary Note 2. Analytical and numerical demonstration for acoustic metagrating with $\xi = 0.6k_0$

In order to test Eq. (4) and Eq. (5), PGM with $\xi = 0.6k_0$ is used for further discussion. Accordingly, the maximum diffraction order is $N = -3$ and the equi-frequency contours of all possible diffraction orders of propagation wave (PW) are shown in Fig. S1(a), where the black dashed circle represents the equi-frequency contour of incident wave and the other solid circles are equi-frequency contours of the corresponding reflected /transmitted waves. If the PGM is composed of five unit cells, i.e., $m = 5$, based on the theoretical prediction, it is mainly transmitted wave of the *n*=1 order for $\theta_{\text{in}} \in \left[-90^\circ, 23.6^\circ\right]$, where $\theta_1 = 23.6^\circ$ is the critical angle of GSL (see the green arrow in Fig. S1(a)). For $\theta_{\text{in}} \in \left[23.6^\circ, 53.2^\circ\right]$, where $\theta_{-3} = 53.2^\circ$ is the critical angle of the $n = -3$ order (see the red arrow Fig. S1(a)), the $n = -2$ order is the maximum diffraction order and the propagation number is $L = 3$. Accordingly, there is mainly a transmitted wave of the $n = -2$ order, for example, see the blue arrows in Fig. S1(a), where the solid blue arrow in the right side denotes the transmitted direction. For $\theta_{\text{in}} \in \left[53.2^\circ, 90^\circ\right]$, the maximum diffraction order turns into the $n = -3$ order, then the propagation number is $L = 2$, bringing about a reflected wave of the $n = -3$ order in dominant. To verify above discussion, we analytically and numerically show the transmission and reflection of the corresponding diffraction orders in Fig. S1(b) and Fig. S1(c), respectively, where the periodic length and thickness of GIM are $p = 5\lambda_0 / 3$ and $h = \lambda_0$ and the effective refractive index of each unit cell is $\rho_j = n_j = 1 + (j-1)\lambda / mh$. The numerical results are obtained from COMSOL MULTIPHYSICS and the analytical results are calculated from the above Coupled Mode Theory. Although there is a little deviation between analytical and numerical results for the steep incident angles, but the variation tendencies of transmission and reflection of these diffraction orders are almost consistent. In particular, there are indeed the transmission order of $n = -2$ in dominant for $\theta_{\text{in}} \in \left[23.6^\circ, 53.2^\circ\right]$ (see the blue data in Fig. S1(b)) and the reflection order of $n = -3$ in dominant for $\theta_{\text{in}} \in \left[53.2^\circ, 90^\circ\right]$ (see the red data in Fig. S1(c)). However, for the incident angle close to $\pm 90^\circ$, the specular reflection is stronger (see black data in Fig. S1(c)), giving arise to lower coupling efficiency for the transmission of the $n = 1$ order and the reflection of the $n = -3$ order.

In addition, when the PGM with $\xi = 0.6k_0$ is constituted by six unit cells ($m = 6$), the equi-frequency contour is identical with that in Fig. S1. Based on our theory summarized in Eq. (4) and Eq. (5), the transmission and reflection of the higher diffraction orders will reverse owing to the change of propagation number. For example, the propagation number of the $n = -2$ order will turn into $L = 4$, which can lead to a reflected wave of the $n = -2$ order in dominant, e.g., see the blue arrows in Fig. S2(a). Accordingly, the propagation number of the $n = -3$ order in PGM with $m = 6$ is $L = 3$, which

results into a transmitted wave of the $n=-3$ order in dominant. Theses theoretical predications are well verified by the analytical and numerical results shown in Fig. S2(b) and Fig. S2(c). Owing to the odevity transition of the propagation number achieved by the change of the number of the unit cells, the transmission and reflection of higher order diffraction will transfer with each other (see Fig. S1 and Fig. S2).

Supplementary Note 3. Design detail and experimental demonstration for acoustic metagrating ($\xi = k_0$) with m=3

To achieve a PGM ($m = 3$) with $\xi = k_0$, we use zigzag microstructures to obtain the corresponding phase shift. There are 0, 6, 10 building blocks in cell-1, cell-2 and cell-3, and these blocks with identical heights are separated by the same distance and (see Fig. S3(a)). By adding more blocks (see the cells from bottom to top), more space will be coiled up to achieve a higher effective refractive index. One can properly adjust the parameters of these blocks to obtain desirable phase shifts. To realize $\xi = k_0$, the phase difference in two 6adjacent cells is required with $\Delta\phi = 2\pi / 3$. The designed parameters are listed in Fig. S3(b), and the corresponding phase shift and transmission of the three unit cells are shown in Fig. S3(c), where the transmissions are almost unity with the phase difference of $\Delta\phi = 2\pi / 3$.

Figure S4 are the numerically simulated (upper) and experimentally measured (lower) acoustic pressure field for the designed PGM ($m = 3$) with $\xi = k_0$. The corresponding results in Fig. S4(a-d) are the cases of the incident angles equal to $\theta_{\text{in}} = 0^{\circ}$, 15°, 45° and 60°. In each plot, the left side is the field pattern of the reflected wave and the right side is the field pattern of the transmitted wave. The numerical and experimental results agree with each other and there are mainly reflected wave following the diffraction order of the $n = -1$ order, which well verifies our theoretical predication. In addition, for the case of normal incidence in Fig. S4(a), we can see that the diffraction wave of the $n = 0$ order is mainly a transmitted wave caused by the odd propagation number (i.e., $L = 3$), which also confirms our theory.

Supplementary Note 4. Design detail and experimental demonstration for acoustic metagrating ($\xi = k_0$) with m=4

Similarly, we also employ zigzag microstructures to realize a PGM ($m = 4$) with $\xi = k_0$. There are 0, 6, 8, 10 building blocks in cell-1, cell-2, cell-3 and cell-4, and all the blocks in each cell are equipped with identical distance and height (see Fig. S5(a)). More space (see the cells from bottom to top) is coiled up to achieve a larger phase shift. By properly adjust the parameters of these blocks, the phase difference of $\Delta\phi = \pi / 2$ in two adjacent cells can be realized and the designed parameters are listed in Fig. S5(b). The corresponding phase shift and transmission of the four unit cells are displayed in Fig. S5(c), we can see that the transmissions are almost unity with the phase difference of $\Delta\phi = \pi / 2$.

Figure S6 are the numerically simulated (upper) and experimentally measured (lower) acoustic pressure field for the designed PGM ($m = 4$) with $\xi = k_0$, where Fig. S6(a-d) are the cases of the incident angles equal to $\theta_{\text{in}} = 0^\circ$, 15°, 45° and 60°. In each plot, the left side is the field pattern of the reflected wave and the right side is the field pattern of the transmitted wave. The numerical results agree with the experimental results and there are mainly transmitted wave following the $n = -1$ order, which well validates our theoretical predication. In addition, for the normal incidence shown in Fig. S6(a), we can find that the diffraction wave of the $n = 0$ order is mainly a reflected wave owning to even propagation number (i.e., $L = 4$), which is opposite with that in Fig. S5(a).

Supplementary Note 5. Parity-dependent transmission and reflection from wave optics

In fact, the formula in Eq. (S11)-(S14) is quite complex to reveal underlying physics. In order to uncover the diffraction mechanism of even/odd-dependence, now we consider a simplified case, that is the PGM with $\xi > k_0$, in which such even/odd-dependent diffraction phenomena also can be seen for normal incidence. Specifically, based on Eq. (4) and Eq. (5) in main text, when *m* is odd, nearly perfect transmission happens; while *m* is even, nearly perfect reflection occurs. Here only three orders, i.e., *n*=0, -1, and 1 are enough for precisely calculating the diffraction efficiency, and for simplicity we assume *w*=*a*. Then some terms in Eq. (S11)-Eq. (S14) are reduced as,

$$f = w/p = 1/m;$$

$$\alpha_0 = 0,\ \alpha_1 = G,\ \alpha_{-1} = -G;$$

$$g_0 = 1,\ g = g_1 = g_{-1} = \mathrm{Sinc}[Gw/2];$$

$$k_z^0 = k_0,\ k_z = k_z^1 = k_z^{-1} = \sqrt{k_0^2 - G^2}\ .$$

With these formula, the Eq. (S11)-Eq. (S14) become

$$(1+r_0) + gr_1\exp(iGx_j) + gr_{-1}\exp(-iGx_j) = a_j + b_j u_j, \quad (j = 1, 2, ..., m) \tag{S15}$$

$$t_0 + gt_1\exp(iGx_j) + gt_{-1}\exp(-iGx_j) = a_j u_j + b_j, \quad (j = 1, 2, ..., m) \tag{S16}$$

$$(1-r_0) = f\sum_{j=1}^{m}(a_j - b_j u_j), \tag{S17}$$

$$r_1 = -\frac{k_0 gf}{k_z}\sum_{j=1}^{m}(a_j - b_j u_j)\exp(-iGx_j), \tag{S18}$$

$$r_{-1} = -\frac{k_0 gf}{k_z}\sum_{j=1}^{m}(a_j - b_j u_j)\exp(iGx_j), \tag{S19}$$

$$t_0 = f\sum_{j=1}^{m}(a_j u_j - b_j), \tag{S20}$$

$$t_1 = \frac{k_0 fg}{k_z}\sum_{j=1}^{m}(a_j u_j - b_j)\exp(-iGx_j), \tag{S21}$$

$$t_{-1} = \frac{k_0 fg}{k_z}\sum_{i=j}^{m}(a_j u_j - b_j)\exp(iGx_j). \tag{S22}$$

Note that $u_j = \exp(ik_j h) = \exp(i\phi_j)$ with $\phi_j = 2\pi j/m$ and $x_j = (j-1)a$, which gives rise to $\exp(-iGx_j) = \exp[-i(j-1)Ga] = u_j^*\exp(i\phi_0)$, where $\phi_0 = k_0 h$. Thereby, the Eq. (S18), Eq. (S19), Eq. (S21) and Eq. (S22) can be further expressed as,

$$r_1 = -\frac{gf}{\gamma}\exp(i\phi_0)\sum_{j=1}^{m}a_j u_j^*, \tag{S23}$$

$$r_{-1} = -\frac{gf}{\gamma}\exp(-i\phi_0)(mt_0 - \sum_{j=1}^{m} b_j u_j u_j) \,, \tag{S24}$$

$$t_1 = \frac{fg}{\gamma}\exp(i\phi_0)(m - \sum_{j=1}^{m} b_j u_j^*) \,, \tag{S25}$$

$$t_{-1} = \frac{fg}{\gamma}\exp(-i\phi_0)(\sum_{i=j}^{m} a_j u_j u_j - mr_0) \,, \tag{S26}$$

where $\gamma = \sqrt{1-(G/k_0)^2}$. When j changes from 1 to m, the phase $\phi = Gx_j$ from $\exp(-iGx_j)$ and $\exp(iGx_j)$ can cover 2π with equal phase difference of $\phi_{j+1} - \phi_j = 2\pi/m$, which leads to $\sum_{j=1}^{m}\exp(iGx_j) = 0$ and $\sum_{j=1}^{m}\exp(-iGx_j) = 0$. Let us sum up m groups of Eqs. (S15) or Eqs. (S16) by considering j=1 to j=m, then the result after integrating Eq. (S15) is written as,

$$m(1+r_0) = \sum_{j=1}^{m} a_j + b_j u_j \,. \tag{S27}$$

By combining Eq. (S17) and Eq. (S27), we can further obtain,

$$r_0 = \frac{1}{m}\sum_{j=1}^{m} b_j u_j \,, \tag{S28a}$$

$$m = \sum_{j=1}^{m} a_j \,. \tag{S28b}$$

Similarly, the result after integrating Eq. (S16) produces

$$mt_0 = \sum_{j=1}^{m} a_j u_j + b_j \,, \tag{S29}$$

and combining Eq. (S20), we also get,

$$t_0 = \frac{1}{m}\sum_{j=1}^{m} a_j u_j \,, \tag{S30a}$$

$$\sum_{j=1}^{m} b_j = 0 \,. \tag{S30b}$$

Box 1. Check the results of Eq. (S28b) and Eq. (S30b).

Eq. (S28b) and Eq. (S30b) are quite interesting results. Although in all analysis, the matched impedances in each slits are used to reduce the reflection, the Eq. S30b tells us that the reflection component from each slit contributed by the coefficient b_j is not always zero or near zero, but their sum are exactly zero. Similarly, the transmission component from each slit contributed by the coefficient a_j is not always untiy or near unity, but their sum are exactly integer number *m*, which is the number of unit cells in a supercell. To further confirm these interesting results, we employ Eq. (S11)-(S15) to figure out both coefficients of a_j and b_j strictly. Here we take *m*=3 and *m*=4 for example, and the used parameters are the same as these in main text. The following shows the calculated data.

1) *m*=3

For a and b coefficients:

$$\begin{cases} b_1 = -0.5433 + 0.8495i \\ b_2 = -0.4553 - 0.8903i \\ b_3 = 0.9987 + 0.0507i \end{cases} \Rightarrow \sum b_i = 0 \,, \quad \begin{cases} a_1 = 0.5903 - 0.9122i \\ a_2 = 0.4148 + 0.8108i \\ a_3 = 1.9948 + 0.1014i \end{cases} \Rightarrow \sum a_i = 3,$$

2) *m*=4

$$\begin{cases} b_1 = 1.78519 - 0.3851i \\ b_2 = -0.7969 + 0.1719i \\ b_3 = -1.78519 + 0.3851i \\ b_4 = 0.796989 - 0.1719i \end{cases} \Rightarrow \sum b_i = 0 \,, \quad \begin{cases} a_1 = 1.6676 - 0.7444i \\ a_2 = 0.3323 + 0.7444i \\ a_3 = 1.6676 - 0.7444i \\ a_4 = 0.3323 + 0.7444i \end{cases} \Rightarrow \sum a_i = 4 \,.$$

Furthermore, by multiplying $\exp(-iGx_j)$ in both sides of Eq. (S15) or Eq. (S16), *m* groups of Eqs. (S15) or Eqs. (S16) are summed up by considering *j* from 1 to *m*, and then we have,

$$r_1 = \frac{1}{mg}\exp(i\phi_0)\sum_{j=1}^{m} a_j u_j^* \,, \tag{S31}$$

$$r_{-1} = \frac{1}{mg}\exp(-i\phi_0)(mt_0 + \sum_{j=1}^{m} b_j u_j u_j) \,, \tag{S32}$$

$$t_1 = \frac{1}{mg}\exp(i\phi_0)(m + \sum_{j=1}^{m} b_j u_j^*) \,, \tag{S33}$$

$$t_{-1} = \frac{1}{mg}\exp(-i\phi_0)\left(mr_0 + \sum a_j u_j u_j\right) . \tag{S34}$$

Comparing Eq. (S23)-(S26) with Eq. (S31)-(S34), we can know that

$$r_1 = 0 \,, \;\; r_{-1} = \frac{2g}{g^2-\gamma}\exp(-i\phi_0)t_0 \,, \;\; t_1 = \frac{2g}{g^2+\gamma}\exp(i\phi_0) \,, \;\; t_{-1} = \frac{2g}{g^2-\gamma}\exp(-i\phi_0)r_0 \,. \tag{S35}$$

With these relationships, Eqs. (S15-S22) reduce to the following simple forms,

$$(1+r_0) + gr_{-1}\exp(-iGx_j) = a_j + b_j u_j \,, \tag{S36}$$

$$t_0 + gt_1\exp(iGx_j) + gt_{-1}\exp(-iGx_j) = a_j u_j + b_j \,, \tag{S37}$$

$$(1-r_0)=f\sum_{j=1}^{m}(a_j-b_ju_j)\,, \tag{S38}$$

$$t_0=f\sum_{j=1}^{m}(a_ju_j-b_j)\,, \tag{S39}$$

Based on Eq. (S36) and Eq. (S37), we get,

$$a_j=-\frac{A_j-B_ju_j}{u_j^2-1} \quad \text{and} \quad b_j=-\frac{B_j-A_ju_j}{u_j^2-1}\,, \tag{S40}$$

where $A_j=(1+r_0)+\tilde{g}_1t_0u_j^*$ and $B_j=t_0+\tilde{g}_{-1}u_j+r_0\tilde{g}_1u_j^*$, with $\tilde{g}_\sigma=2g^2/(g^2-\sigma\gamma)$. Because g is a real and γ is a complex, then $\tilde{g}_1=\tilde{g}_{-1}^*$. By submitting Eq. (S40) into Eq. (S38) and Eq. (S39), then we can get two equations only involving r_0 and t_0, i.e.,

$$r_0(m-\sum_{j=1}^{m}\frac{u_j^2-\tilde{g}_1}{u_j^2-1})+t_0(1-\tilde{g}_1)\sum_{j=1}^{m}\frac{u_j}{u_j^2-1}=(1-\tilde{g}_{-1})\sum_{j=1}^{m}\frac{u_j^2}{u_j^2-1}\,, \tag{S41}$$

$$r_0(1-\tilde{g}_1)\sum_{j=1}^{m}\frac{u_j}{u_j^2-1}+t_0(m-\sum_{j=1}^{m}\frac{u_j^2-\tilde{g}_1}{u_j^2-1})=\sum_{j=1}^{m}\frac{\tilde{g}_{-1}u_j^3-u_j}{u_j^2-1}\,. \tag{S42}$$

By solving Eq. (S41) and (S42), we can get,

$$r_0=\frac{c_1\zeta_1-c_2\zeta_2}{\zeta_1^2-\zeta_2^2}\,,\quad t_0=\frac{c_1\zeta_2-c_2\zeta_1}{\zeta_1^2-\zeta_2^2} \tag{S43}$$

where

$$\zeta_1=-(1-\tilde{g}_1)\sum_{j=1}^{m}\frac{1}{u_j^2-1}\,,\quad \zeta_2=(1-\tilde{g}_1)\sum_{j=1}^{m}\frac{u_j}{u_j^2-1}\,;$$

$$c_1=(1-\tilde{g}_{-1})\sum_{j=1}^{m}\frac{u_j^2}{u_j^2-1}\,,\quad c_2=\sum_{j=1}^{m}\frac{\tilde{g}_{-1}u_j^3-u_j}{u_j^2-1}\,.$$

Based on $\tilde{g}_1=\tilde{g}_{-1}^*$ and $u_ju_j^*=1$, we can find the following relationships,

$$\zeta_1=c_1^* \quad \text{and} \quad \zeta_2=c_2^*\,. \tag{S44}$$

Using these relations, a simple formula for the reflection and transmission can be obtained,

$$r_0=\frac{|\zeta_1|^2-|\zeta_2|^2}{\zeta_1^2-\zeta_2^2} \quad \text{and} \quad t_0=\frac{\zeta_1\zeta_2^*-\zeta_2\zeta_1^*}{\zeta_1^2-\zeta_2^2}\,. \tag{S45}$$

From Eq. (S45), we can know that the reflection and transmission are only determined by two factors: (*i*) one is the coefficient $\tilde{g}_1$, which is related to the geometry structure of PGM. For a fixed configuration, $\tilde{g}_1$ is a constant; (*ii*) the other one is the introduced abrupt phase $u_j=\exp(i\phi_j)$, which brings about two elements,

$$Y_1=\sum_{j=1}^{m}\frac{1}{u_j^2-1}=\sum_{j=1}^{m}\frac{1}{\exp(2i\phi_j)-1} \quad \text{and} \quad Y_2=\sum_{j=1}^{m}\frac{\exp(i\phi_j)}{\exp(2i\phi_j)-1}\,. \tag{S46}$$

Obviously, Y_1 and Y_2 are highly dependent of the phase distribution $\phi_j = 2\pi j/m$ in *u*-complex plane. When *m* is even, the discrete distribution of phase ϕ_j in *u*-plane is symmetric, namely, $u_j = -u_{m/2+j}$ $(j \le m/2)$, which leads to $Y_2 = 0$. When *m* is odd, the symmetry of phase distribution is broken; but we can find the following relationship, $\phi_j + \phi_{m-j} = 2\pi$ $(j \in [1,(m-1)/2])$, which leads to $u_j = -u_{m-j}$. As a result, $Y_1 = \sum_{j=1}^{m} \frac{1}{u_j^2 - 1} = \frac{1}{\exp(2i\phi_m) - 1}$ and $Y_2 = \sum_{j=1}^{m} \frac{\exp(i\phi_j)}{\exp(2i\phi_j) - 1} = \frac{\exp(i\phi_m)}{\exp(2i\phi_m) - 1}$, i.e., $|Y_1| = |Y_2|$. With these relations, Eq. (S46) can be further simplified as:

(i) when *m* is even, $Y_2 = 0$ and $\zeta_2 = 0$, then

$$\boxed{r_0 = \frac{|\zeta_1|^2 - |\zeta_2|^2}{\zeta_1^2 - \zeta_2^2} = \exp(-i\varphi_R)\,;\ t_0 = \frac{\zeta_1\zeta_2^* - \zeta_2\zeta_1^*}{\zeta_1^2 - \zeta_2^2} = 0}, \tag{S47}$$

where $\varphi_R = 2\mathrm{Arg}[\zeta_1]$ is the reflected phase.

(ii) when *m* is odd, $|Y_1| = |Y_2|$ and $|\zeta_1| = |\zeta_2|$, then

$$\boxed{r_0 = \frac{|\zeta_1|^2 - |\zeta_2|^2}{\zeta_1^2 - \zeta_2^2} = 0\,;\ t_0 = \frac{\zeta_1\zeta_2^* - \zeta_2\zeta_1^*}{\zeta_1^2 - \zeta_2^2} = \frac{\zeta_2^*}{\zeta_1} = \exp(-i\varphi_T)}. \tag{S48}$$

where $\varphi_T = \mathrm{Arg}[\zeta_2] + \mathrm{Arg}[\zeta_1]$ is the transmitted phase.

Supplementary Note 6. The band performance of a designed PGM

As the phase gradient unit cells are designed at the targeted frequency, a well-designed metagrating only works in a narrowband. If the working frequency deviates from the targeted one, the required phase and transmission profiles in these individual unit cells are also compromised. As a result, the transmission/reflection efficiencies of the desired diffraction orders will reduce owing to the generation of other undesired diffraction orders. Therefore, the major effects are not completely lost for a slight frequency shift.

For example, we show the transmission and reflection of the designed metagrating with m=3 at other wavelengths. The required phase and transmission profiles are maintained approximately from 0.95λ_0 to 1.1 λ_0 (see Fig. S7(a) and 7(b)), the desired performance of the device can be preserved, e.g., see the result in Fig. S7(e). For a bigger change in wavelength (e.g., 0.9 λ_0 or 1.2 λ_0), both the phase and transmission profiles deviate from the desired results, and the performance further reduces (see the Fig. S7 (d) and 7(f)). In the smaller wavelength regime (e.g., 0.8λ_0), transmission profiles are good, but phase profiles deviate from the desired results, leading to a compromised performance (see the Fig. S7 (c)).

It is difficult to quantify the device using a certain quality factor, as the practical functionality of the device is highly dependent on the monolithic response of these designed unit cells. This response usually has intricate phase and transmission response in the spectrum, and could vary for different types of designs. Therefore, the performance of the designed device only can be numerically/experimentally tested case by case, which is quite common in the subject of phase-gradient metasurfaces.
